# Honors Accumulating

**Published:** 1875-07

**Authors:** 


Honors Accumulating.—Dr. J. G. Thomas, of Savannah, Ga., was brought forward by his friends last fall as a candidate for the State Legislature, and was elected by a handsome majority. At the winter session he took his seat in the House, and soon showed himself to be an able representative of his constituency as well as of the medical profession. By his influence and exertions a law was passed, which we give to our readers, establishing a State Board of Health. At the April meeting of the Medical Association of Georgia Dr. Thomas was made its president for the ensuing year. Upon the organization of the Health Board in June he was elected to the presidency of that body, and now the newspapers bring forward his name as a candidate for the next governorship of Georgia. Truly do the honors thicken around him.

---

It is proposed to erect at Hartford a bronze statue and monument to commemorate Horace Wells as the discoverer of anæsthesia. Those who desire to make contributions to this object should remit to Dr. G. W. Russell, Treasurer, Hartford, Conn.

